# How early to start: what do observational data suggest?

**DOI:** 10.1186/1758-2652-13-S4-O34

**Published:** 2010-11-08

**Authors:** J Sterne

**Affiliations:** 1 University of Bristol, Department of Social Medicine, Bristol, UK

## 

National and international guidelines on antiretroviral therapy for HIV infection recently changed to recommend earlier treatment, following the publication during 2009 of two collaborative analyses of HIV cohort studies [1,2]. These studies used different methodologies, and reached different conclusions about the benefits of very early treatment. I will describe their methods, and possible reasons for differences in their results.
